# Overcoming Resistance to Checkpoint Inhibitors: Natural Killer Cells in Non-Small Cell Lung Cancer

**DOI:** 10.3389/fonc.2022.886440

**Published:** 2022-05-31

**Authors:** Maria Gemelli, Douglas M. Noonan, Valentina Carlini, Giuseppe Pelosi, Massimo Barberis, Riccardo Ricotta, Adriana Albini

**Affiliations:** ^1^ Istituto di Ricovero e Cura a Carattere Scientifico (IRCCS) MultiMedica, Milan, Italy; ^2^ Istituto di Ricovero e Cura a Carattere Scientifico (IRCCS) MultiMedica Science and Technology Park, Milan, Italy; ^3^ Immunology and General Pathology Laboratory, Department of Biotechnology and Life Sciences, University of Insubria, Varese, Italy; ^4^ Department of Oncology and Hemato-Oncology, University of Milan, Milan, Italy; ^5^ Department of Pathology, European Institute of Oncology (IEO) Istituto di Ricovero e Cura a Carattere Scientifico (IRCCS), Milan, Italy; ^6^ European Institute of Oncology (IEO) Istituto di Ricovero e Cura a Carattere Scientifico (IRCCS), Milan, Italy

**Keywords:** natural killer, non-small cell lung cancer, tumor microenvironment, checkpoint inhibitors, inflammation, angiogenesis, polarization, resistance

## Abstract

Immune checkpoint inhibitors (ICIs) have revolutionized cancer treatments over the last 10 years, with even increasing indications in many neoplasms. Non-small cell lung cancer (NSCLC) is considered highly immunogenic, and ICIs have found a wide set of applications in this area, in both early and advanced lines of treatment, significantly changing the prognosis of these patients. Unfortunately, not all patients can benefit from the treatment, and resistance to ICIs can develop at any time. In addition to T lymphocytes, which are the major target, a variety of other cells present in the tumor microenvironment (TME) act in a complex cross-talk between tumor, stromal, and immune cells. An imbalance between activating and inhibitory signals can shift TME from an “anti-” to a “pro-tumorigenic” phenotype and vice versa. Natural killer cells (NKs) are able to recognize cancer cells, based on MHC I (self and non-self) and independently from antigen presentation. They represent an important link between innate and adaptive immune responses. Little data are available about the role of pro-inflammatory NKs in NSCLC and how they can influence the response to ICIs. NKs express several ligands of the checkpoint family, such as PD-1, TIGIT, TIM-3, LAG3, CD96, IL1R8, and NKG2A. We and others have shown that TME can also shape NKs, converting them into a pro-tumoral, pro-angiogenic “nurturing” phenotype through “decidualization.” The features of these NKs include expression of CD56, CD9, CD49a, and CXCR3; low CD16; and poor cytotoxicity. During ICI therapy, tumor-infiltrating or associated NKs can respond to the inhibitors or counteract the effect by acting as pro-inflammatory. There is a growing interest in NKs as a promising therapeutic target, as a basis for adoptive therapy and chimeric antigen receptor (CAR)-NK technology. In this review, we analyzed current evidence on NK function in NSCLC, focusing on their possible influence in response to ICI treatment and resistance development, addressing their prognostic and predictive roles and the rationale for exploiting NKs as a tool to overcome resistance in NSCLC, and envisaging a way to repolarize decidual NK (dNK)-like cells in lung cancer.

## 1 Introduction

**Figure 1:**
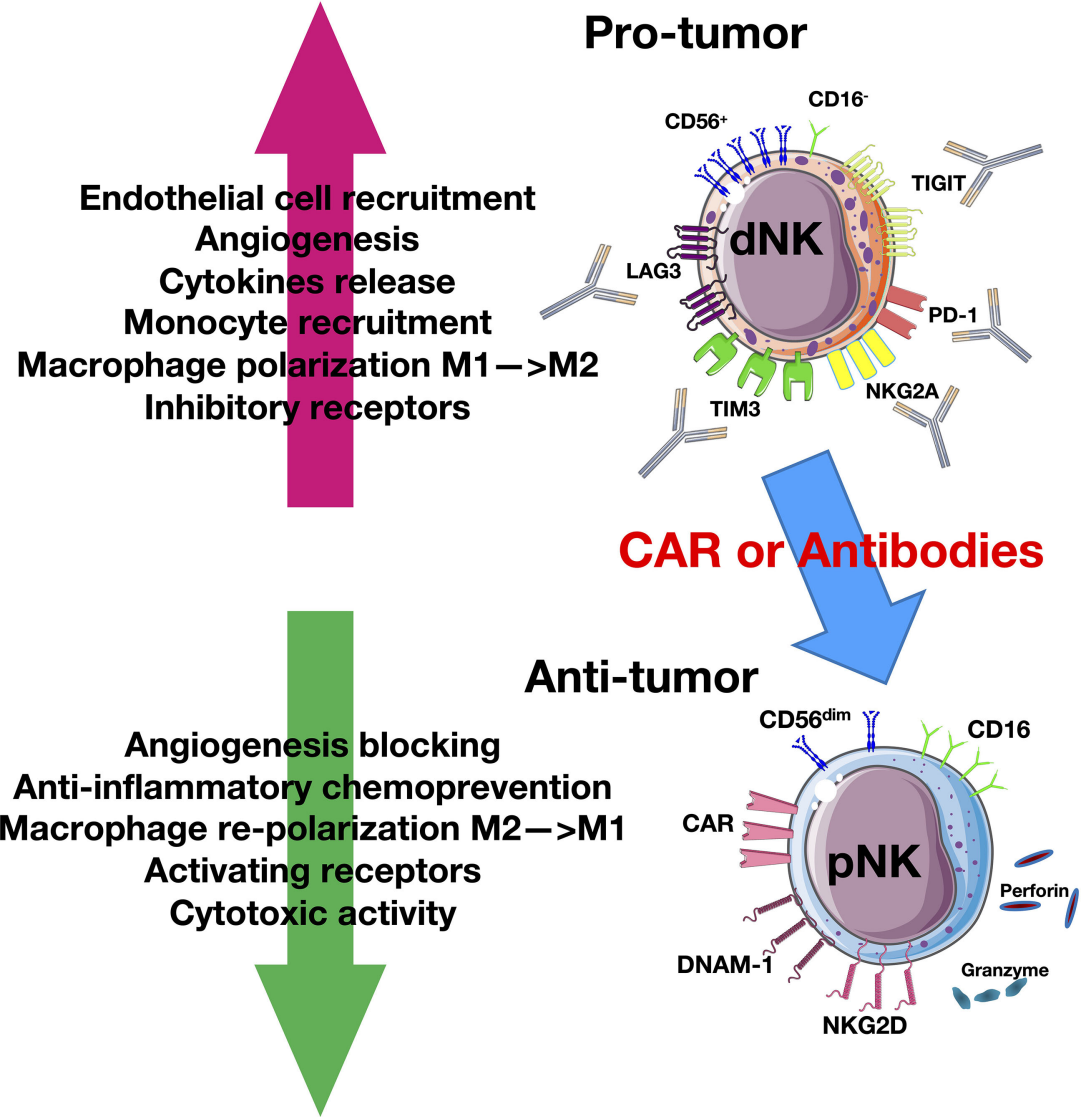
Natural killer cell (NK) plasticity in cancer. Tumor cells and tumor microenvironment (TME) induce a pro-tumor CD56^+^CD16^−^ decidual-like phenotype that expresses inhibitory receptors. The blockade of these receptors or the use of chimeric antigen receptor (CAR) or adaptive therapy can reverse this mechanism by switching NKs into antitumor cytotoxic CD56^dim^CD16^+^ cytotoxic phenotype that can release granzyme and perforin.

Immunotherapy has become a milestone in the treatment of almost all kinds of neoplasms, both solid and hematologic. While chemotherapy is aimed to kill cancer cells, immunotherapy stimulates the immune system to react against tumors (1). The concept of cancer immunotherapy is based on the finding that tumor cells, normally recognized and neutralized by T cells, can develop mechanisms to evade the host’s immune surveillance. Thus, inhibition of negative regulators of T-cell function may increase the activation of the immune system, inducing a subsequent enhancement of antitumor responses as well (1). Great progress has been made from the first attempts with cancer vaccines leading to the approval of the more recent immune checkpoint inhibitors (ICIs). Among these, the first therapeutic molecules to be developed and to have brought a clinical improvement are the anti-cytotoxic T-lymphocyte antigen 4 (CTLA-4), also known as CD152, and the anti-programmed death receptor-1/programmed death ligand-1 (PD-1/PD-L1) antibodies. These agents, alone or in combination, are routinely used in clinical practice for the treatment of many solid tumors, such as lung cancer, urothelial and renal cell carcinoma, head and neck tumors, melanoma, and mismatch repair deficient colon cancer (2). ICIs act by blocking the activation of tumor-induced inhibitory pathways: the first one (anti-CTLA-4) mostly at the early stage of naïve T-cell activation, at the site of antigen presentation in lymph nodes, and the latter (anti-PD-1/PD-L1) at the advanced stage of a T-cell immune response, directly in the tumor microenvironment (TME) (3), as depicted in **Figure 1**. In addition to PD-1/PD-L1 and CTLA-4, other checkpoint molecules such as the T-cell immunoglobulin and mucin domain 3 (TIM-3), lymphocyte activation gene-3 (LAG3), T-cell immunoglobulin and immunoreceptor tyrosine-based inhibitory motif (ITIM) domain (TIGIT), and, more recently, V-domain immunoglobulin suppressor of T-cell activation (VISTA) have been explored as potential targets for the development of new agents for cancer immunotherapy (4). Non-small cell lung cancer (NSCLC), the most common cause of cancer-related death worldwide, is considered a highly immunogenic neoplasm. ICIs have found a wide range of applications in this oncologic field, in both early and advanced lines of treatment, dramatically changing the prognosis of these patients in many cases (5–12). Unfortunately, not all patients can benefit from this treatment, and resistance can occur even in individuals who were previously responsive (13, 14). Several explanations have been provided for the lack of efficacy of ICIs, one of which is related to the low presence of T lymphocytes to be reactivated by targeting the immune checkpoints. The fine balance between activating and suppressing signals of the immune system plays a pivotal role in promoting or, conversely, counteracting cancer onset and progression. Tumor-infiltrating T lymphocytes have emerged as important prognostic and predictive factors in many types of cancer. In particular, the percentage of CD8^+^ T lymphocytes as well as the CD4^+^/CD8^+^ ratio and the polarization toward an anti-cancer T-helper response (Th1 vs. Th2), seem to correlate with better prognosis and improved response to ICIs in melanoma, breast, and lung cancers (15–17). Another reason lies in the complex interaction existing between innate immunity, stromal, and tumor cells in TME, which is crucial in regulating tumor formation, growth, invasion, and metastasis. The importance of tumor-associated macrophages (TAMs) in tumor response has been recently understood. Some preliminary observations propose that polarization toward a pro-tumorigenic M2 phenotype correlates with worse prognosis and increased risk of recurrence after resection in different types of cancer, including lung tumors; more recent evidence also suggests a possible negative predictive role for response to ICIs (15, 18). We have been working for a long time on tumor-associated natural killer cells (NK) (TA-NKs). Despite their crucial role in immunity, limited data are evaluable about their role to modulate the response to immunotherapy. In this review, we want to summarize and discuss the data currently available on the behavior of NKs under ICI treatment, their role in resistance to treatment and possible strategies to exploit their function as a therapeutic target, and their potential re-polarization into killers, with a focus on NSCLC.

## 2 Natural Killer Cell Phenotype and Biology

NKs are lymphoid cells of the innate immune system, representing about 20% of total peripheral blood (PB) circulating lymphocytes. Since their discovery in the 1970s, they have aroused growing interest thanks to their potent cytolytic function against tumor cells or virus-infected cells without previous antigen sensitization or immunologic memory (19–21). T-cell immunity requires recognition of specific antigens presented through major histocompatibility complex (MHC) class I and class II proteins by CD8^+^ and CD4^+^ T cells (22, 23), respectively, while NKs recognize as “non-self”, tumor cells that have lost their MHC class I molecules (19, 20). Furthermore, they are primary producers of interferon-gamma (IFN-γ), the most potent stimulus for MHC expression and antigen presentation, acting as a cross-talk between innate and adaptive responses. Recently, NKs have been recategorized as type 1 in the larger family of innate lymphoid cells (ILCs) (24–26).

NKs exert both cytotoxic and regulatory activities. On the one hand, they induce apoptosis and cell death through the release of perforin and granzyme by their intracytoplasmic granules; on the other hand, they orchestrate innate response through the secretion of immunomodulatory soluble factors, such as cytokines and chemokine, which act on hematopoietic cell recruitment and activation (24–28).

Two major immunophenotypic subpopulations of NKs, which differ in morphology and function, have been identified in PB based on the relative expression of the CD56 and CD16 antigen surface markers. The first subset, the CD56^dim^CD16^+^, accounts for 95% of NKs in PB, and, when it comes into contact with virus-infected cells or tumor cells expressing low levels of MHC class I or other ligands, exhibits high cytotoxic activity through perforin and granzyme release. The other subset, the CD56^bright^CD16^−^, represents only 5% of NKs circulating in PB, but it is the majority of NKs in secondary lymphoid tissues and shows low cytotoxic potential and efficient production of cytokines, such as the tumor necrosis factor (TNF)-alpha and the granulocyte macrophage colony-stimulating factor (GM-CSF), both of which play a crucial role in the modulation of an immune response, particularly in chronic inflammation. These cells are considered as a less differentiated form than the “terminally differentiated” CD56^dim^CD16^+^, subtype (29). In fact, when exposed to interleukin (IL)-2, IL-12, and/or IL-15, they can differentiate into granules secreting CD56^dim^CD16^+^ NKs (30–32).

NKs dynamically circulate between organs and the bloodstream to exert their immunosurveillance activity (33, 34). Among organs, the lungs have the highest contents of NKs, mostly the CD56^dim^CD16^+^ subtype (34–37). An analysis performed on NKs isolated from bronchoalveolar lavage of normal lungs underlined that pulmonary NKs are mainly functionally inactive and show a weaker response to immune stimulation, as a consequence of local inhibitory influences (38). However, in response to proper stimuli, such as IL-2, lung NK activity is completely restored. This suggests that pulmonary alveolar macrophages can regulate lung NK activity for the maintenance of physiological homeostasis (38), as the lungs are continuously exposed to novel antigens (38, 39).

The third type of NKs showing a CD56^bright^CD16^−^ phenotype and the expression of killer-cell immunoglobulin-like receptors (KIR) receptor, CD69, and CD49a on the cell surface has been recently described (35, 40, 41). About 15% of tissue-resident NKs in the lungs are CD56^bright^CD16^−^CD49a^+^, with a high ability to secrete IFN-γ (40). This can be considered a “decidual” phenotype (dNK), recapitulating an NK type first described in the decidua and which has a crucial role in the tolerance of the embryo and the spiral artery formation (42–45). The dNK was observed in many cancers, both infiltrating and in PB. In cancer patients, it acts in a “pro-tumorigenic” way, inducing tolerance and proving nurturing function, similar to what happens with the embryo (42).

## 3 Natural Killer Cells in Non-Small Cell Lung Cancer and Modulation by the Tumor Microenvironment

NKs were found to be an important part of the TME in various cancer types, able to modulate the immune response and affect prognosis, particularly in lung cancer (35–37, 40, 41, 46–48). Although these cells normally carry out immune surveillance and have the function of destroying tumor cells, they can also act as tumor-promoting inflammatory leukocytes. This is in large due to the modulation by both the tumor itself and the TME, which is constituted by various immune cells, fibroblasts, extracellular matrix, growth factors, and endothelial and vascular cells: an imbalance between activating and inhibitory signals can determine whether NKs will exert their cytotoxic activity or remain inactive or even become pro-tumor (46). Intratumorally, NKs have a prognostic significance in lung cancer: high NK infiltration was positively correlated with survival rate in patients who underwent surgery in early stages, and in particular, increased NK infiltration was found in squamous cell carcinoma (SCC), in non-smoking patients, and lower-stage tumors (T1–T2 and limited nodal involvement) (46).

In NSCLC patients, NKs were found at the invasive margin of tumor samples (35, 48). Tumor-infiltrating NKs (TI-NKs) in lung cancer are mostly of the CD56^bright^CD16^−^ subset and exhibit low cytotoxic potential as well as high cytokine production capability. They are mainly present in the tumor stroma, particularly in the alveolar and peri-bronchovascular interstitium, without direct interaction with tumor cells, suggesting a major role in the orchestration of the immune response rather than killing effect (36, 48). In contrast, the percentage of cytotoxic CD56^dim^CD16^+^ NKs is lower in lung cancer compared to normal tissue, probably as a result of the modulation by TME, and it is related to MHC class I expression, as it is higher in MHC I-deficient tumors.

TME can directly contribute to the bloodstream recruitment and the accumulation of CD56^bright^CD16^−^ NKs at the tumor site by promoting a switch in chemokine expression. In particular, the number of CD56^bright^ NKs infiltrating NSCLC is correlated with a downregulation of C-X-C Motif Chemokine Ligand (CXCL)2, the chemokine specifically attracting CD56^dim^ NKs, and overexpression of the chemokines preferentially attracting CD56^bright^ NKs, CXCL9, CXCL10, and C-C Motif Chemokine Ligand (CCL)19. These chemokines, through the binding to C-X-C Motif Chemokine Receptor (CXCR)3, promote low-cytotoxic CD56^bright^ NK recruitment, ultimately leading to tumor escape (34). TI-NKs show a deep alteration of their phenotype, with overexpression of CXCR3 receptor and downregulation of CD57 mature NK marker, and have profound defects in their ability to activate granzyme B degranulation and IFN-γ production (49).

Whether the presence of TI-NKs and their tumor-specific characterization affect prognosis and treatment sensitivity is largely unknown. A high proportion of TI-NKs have been associated with longer progression-free survival (PFS) in advanced and resected early-stage NSCLC, in both squamous and adenocarcinoma (50–52). Conversely, in a recent meta-analysis performed on NKs infiltrating solid tumors, including four studies on lung cancer, no correlation was found between the degree of NK infiltration and overall survival (OS) in patients from stage I to IV (53). However, the small sample size, the high variability in methods used for analysis, and the large differences in stages and histological profiles in all these studies make it difficult to draw definitive conclusions. Furthermore, such heterogeneous results might depend on the dual nature of the NKs themselves, since, against all the “dogmas” on terminal differentiation, they can switch from a cytotoxic antitumor activity to an exhausted pro-tumoral function under pressure and modulation of tumor and TME.

TME is composed of a multitude of immune cells, in addition to T and B cells, macrophages, granulocytes, mast cells, fibroblasts and extracellular matrix, secreting growth factors, activating or inhibitory cytokines, and chemokines and proteases, all of which are in dynamic spatial and temporal evolution. An imbalance in cellular and soluble inhibitory factors results in the establishment of a pro-tumoral microenvironment, which in turn supports tumor growth, progression, and resistance. NKs have pleiotropic functions, and given their dual nature between innate and adaptive immunity, TME may deeply affect their function to contrast or to support tumor growth and promote immune escape. As in other observed malignancies, TI-NKs derived from NSCLC displayed an impaired degranulation activity and INF-γ production when exposed to tumor cells than NKs present in normal lung tissue or circulating in the bloodstream (48, 54). Furthermore, T1-NKs produce placental-derived growth factor (PIGF), vascular endothelial growth factor (VEGF), and IL-8/CXCL8, particularly in SCC (37). We found that the CD56^+^CD16^−^ NKs represented the predominant subset in samples from 31 surgically resected NSCLC and a minor subset in samples from adjacent normal lung tissue and PB (37). We also observed that NK supernatants derived from NSCLC samples induced endothelial cell chemotaxis and formation of capillary-like structures *in vitro*, particularly evident in SCC patients and absent in controls (37). Taken together, these data suggest that in NSCLC, and particularly in SCC, NKs act as proangiogenic cells with a mechanism at least in part mediated by transforming growth factor-beta (TGF-beta). TI-NKs infiltrating the tumors have been shown to have a phenotype characterized by CD56^bright^CD16^−^/low CD94/NK group 2 member A (NKG2A)^+^ perforin low (36, 37, 47) and decreased expression of CD337/NK protein (NKp)30, NKp80/KLRF1, CD226/DNAX accessory molecule (DNAM-1), CD16, and CD85j/Ig-like transcript (ILT2) inhibitory receptors. TI-NKs in NSCLC patients show uniformly poor cytotoxicity and acquire a pro-angiogenic dNK-like phenotype, described as VEGF+ CXCL8+ PlGF+ (37, 42, 47). This NK subtype was observed in many cancers, in both infiltrating tumoral tissues and the PB (37, 41, 42, 47). In cancer, these cells lack the ability to kill malignant cells and directly act in a “pro-tumorigenic” way, inducing immune tolerance and providing nurturing function (37, 42, 47), similar to what happens in the embryo.

The production of pro-angiogenic factors not only is limited to TI-NKs but also is observed in PB NKs (tumor-associated NKs (TA-NKs)) (37, 42, 47). TA-NKs present similar phenotypic characteristics compared to TI-NKs (37, 41, 42, 47, 55, 56). The presence of these NKs in PB results in a potent systemic pro-tumorigenic effect even in early-stage small-size carcinomas, especially for the SCC (37, 47). The TME interacts with the immune system and may impair NK activity through different strategies, including the production of inhibitory cytokines, such as TGF-beta and IL-10, the high infiltration of peritumoral monocytes/macrophages, which can induce the polarization of NK toward a pro-tumorigenic phenotype, and the inhibition of natural cytotoxicity receptor (NCR) expression, mainly NKp30, NKp44, and NKp46 (57–59). Here we describe the main mechanisms involved.

### 3.1 Decidual Natural Killer Cells

As reported above, dNK cells are a third NK subset that has recently been described and differs from the PB subset at both functional and phenotypical levels. They show a CD56^bright^CD16^−^ phenotype, a characteristic expression profile of KIR receptors, various chemokine receptors, and tissue residency markers CD9 and CD49a on the cell surface (43–45). CD9 is a member of the tetraspanin family, which is associated with different integrin adhesion receptors and modulates cell migration, invasion, and adhesion. CD9 is upregulated by TGF-beta (60) and is also characteristic of exosomes (61) CD49a constitutes the alpha-subunit of the alpha1beta1 integrin receptor (VLA1), which binds collagen IV present in basement membranes and is involved in regulating cell cytotoxic activity, migration, and adhesion (43).

This NK subtype was first identified in the as decidual placenta and uterus and for this reason called dNKs (43, 62). dNKs are highly proangiogenic and have a fundamental function in decidual vascularization and spiral artery formation, through the secretion of proangiogenic cytokines like PlGF, angiogenin, CXCL8, VEGF, and angiopoietins 1 and 2 (44, 63–66). When added to tumor cell xenografts, dNK cells can stimulate neoangiogenesis and tumor growth (63). dNKs play also an important role in maintaining immune homeostasis: acting as an immunosuppressant and losing their killing ability, they create a microenvironment protected by the recognition of the immune system and capable of tolerating the growth of the embryo (43–45).

Similar mechanisms occur in tumors; cancer cells can shape the TME, converting immune cells from a cytolytic to a tolerant and nurturing phenotype (42). dNK cells with proangiogenic decidual features have been described in lung cancer and other tumors, such as colorectal and prostate cancers (42). The dNK cell decidual marker CD9 is expressed by TI-NKs of melanoma, colorectal cancer, breast cancer, and glioblastoma (41, 42, 55, 56, 67–70). The chemokine receptor CXCR3, another dNK marker, is expressed in TI-NKs of colorectal cancer, breast cancer, melanoma, and glioblastoma (42, 49, 67–69), while CXCR4 in NK is upregulated in neuroblastoma and prostate cancer (42, 71). TA-NKs express CD9 and CD49^+^ in NSCLC, prostate cancer, and melanoma (41, 42, 56, 69), and CXCR4 is present in TA-NKs of prostate cancer (56). TME induces accumulation of CD56^bright^CD16^−^ poorly cytotoxic NKs, promotes their survivorship and NK decidualization, and reprograms them to resume embryonic activity finalized to tumor immune escape and growth (42). TME can exert this function through the release of a large number of proangiogenic factors, like adenosine (ADO), hypoxia, prostaglandin E2 (PGE-2), glycodelin-A (GdA), HLA-G, and galectin-1 (42). Among these molecules, TGF-beta seems to be the most potent cytokine inducing immune response downregulation and NK decidualization, and it is found to be upregulated in many tumor types (60, 72, 73).

CD56^bright^CD16^−^ NKs represent the predominant subset in resected NSCLC and show proangiogenic features, such as VEGF, P1GF, and IL-8 secretion, particularly evident in SCC (37, 42, 47). In our previous publications, we showed that supernatants derived from TI-NKs and TA-NKs can induce endothelial cell chemotaxis and capillary formation *in vitro* (37). NKs expressing decidual-like markers, such as CD49a and CD9, have also been found in pleural effusion from primary and metastatic tumors, including lung cancer. These cells showed compromised degranulation activity and IFN-γ production and enhanced VEGF secretion, which was partially restored with the addition of IL-2 (37, 74).

Our data suggest that tissue inhibitors of metalloproteases (TIMPs) might counteract cancer-induced NK polarization, by restoring the expression of activation markers like NKG2D and reducing the expression of exhaustion markers such as CD9, CD49a, and the T-cell immunoglobulin and mucin-domain containing-3 (TIM-3) (75). Taken together, these and many other results suggest an important role for NK polarization in tumor growth and invasion, including in NSCLC. Understanding these mechanisms is fundamental for the development of new therapeutic strategies. A blockade of decidualization could constitute a new therapeutic target, not only in lung cancer but also in other malignancies sharing this phenomenon.

### 3.2 Activating and Inhibitory Receptors

A mechanism by which TME may shape NKs into a non-cytotoxic phenotype is the reduction of activating NK receptors and the induction of inhibitory receptors on the cell surface. The tolerance toward self-healthy cells is mediated by HLA molecules that bind to inhibitory HLA NK receptors, mainly KIRs and CD94/NKG2A, mitigating NK cytotoxic ability (36, 48). NKG2A is an inhibitory member of the NKG2 family and is expressed on CD56^high^ NKs (76, 77). The non-classical MHC class I molecule HLA-E is the major ligand of NKG2A^−^CD94 (76–78). High NKG2A expression on the cell surface is a marker of NK exhaustion and correlates with a worse prognosis (76–79). This goes in parallel with the downregulation of NCRs such as NKp30, NKp44, and NKp46, by a mechanism that is supposed to depend on cell-to-cell contact (76–79). Upregulation of inhibitory NK receptors occurs in cancer (76–78). As for “decidualization,” TGF-beta is the most potent stimulus to induce upregulation of inhibitory T-cell receptors (TCRs) and downregulation of the activating ones on NKs (60, 72, 73). Lung cancer produces a high amount of TGF-beta, and the circulating levels of this factor correlate with prognosis (48) and diagnostic effects for patients with early-stage NSCLC (80).

Inhibitory checkpoints have an important role in maintaining homeostasis and usually are not expressed by resting NKs, but in cancer and other pathological condition, their production is induced by the interaction of ligands released by tumor cells, to allow immune escape.

Among these, TIGIT is an important co-inhibitory receptor of the immunoglobulin superfamily expressed by NKs (81). Together with CD96, TIGIT binds to CD155 and CD112 resulting in NK and T-cell inhibition (82). Like TIGIT, TIM-3 has been investigated as a marker of T-cell exhaustion because it is frequently co-expressed with PD-1 and has recently been found overexpressed in circulating NKs of advanced lung cancer (83). Moreover, TIM-3 is overexpressed in CD3^−^CD56^+^ NKs, and it is higher in patients with advanced lung adenocarcinoma (nodal involvement or T3–T4); this overexpression is correlated with shorter OS. Interestingly, blocking TIM-3 alone or in combination with an anti-PD1 may reverse the NK exhaustion (84).

Beyond a well-established regulatory role in T-cell activation, overexpression of LAG3 was associated with decreased NK function in mouse models. However, this has not been confirmed in humans, so further studies might focus on T-cell regulation for LAG3 rather than NK function (85–87).

NK activation is partially controlled by KIRs upon binding with their ligands, primarily the HLA-C molecules. KIRs are a large family that comprehends several inhibitory receptors, which bind to different allotypes of MHC complexes. Through this binding, KIRs activate intracellular inhibitory signals that prevent NK activation (88). The importance of KIR inhibition has been demonstrated in acute myeloid leukemia (AML) patients in whom allogeneic transplantation of stem cells having a mismatch between KIRs on donor NKs and recipient MHC class I molecules was likely to reactivate NK antitumor function, leading to improved relapse-free survival and OS. The results suggest that blocking the interaction between KIRs and MHC class I results in NK activation and subsequent eradication of the residual leukemia clones (89).

PD-1 is one of the most important immune checkpoints, with relevant clinical applications. PD-1 was first described in T cells, but it is also expressed in NKs (90–92). Like other checkpoint regulators, its primary role is to maintain cell homeostasis. However, cancer cells can express their ligand (PD-L1) and, together with other inhibitory immune cells, like the regulatory T cells (Tregs), can release TGF-beta to induce PD-1 expression on NKs, thereby escaping the immune response (90–92). Furthermore, PD-L1 expressing circulating epithelial tumor cells CETCs have been detected in 82% of lung cancer patients. PD-L1 positive CETCs could be a potential biomarker to select patients for treatment with PD-1/PD-L1 inhibitors, and may be a direct target of anticancer treatment (93).

All these receptors are currently under investigation in clinical trials as targets.

### 3.3 Alterations of Natural Killer Cell Metabolism

NK metabolism might be impaired in TI-NKs, limiting NK cytotoxic activity. TME plays a major role even in this context, as it consumes a large number of nutrients, such as glucose and glutamine, and releases TGF-beta, which in turn reduces NK glycolysis and oxidative phosphorylation, decreasing NK activity (94). Inhibition of the TGF-beta pathway restores NK metabolism, underlying its importance in TME regulation (94). The enzyme fructose-1,6-biphosphates 1 (FBP1) is upregulated by TGF-beta during cancer progression, resulting in functional NK impairment (95). An FBP1 inhibitor has recently been developed and showed preclinical evidence of restoring NK function (95). In addition to low glucose concentrations, TME is characterized by other conditions that can decrease NK function, like hypoxia and acidic pH (96). Moreover, hypoxia can reduce NK surface expression of activating receptors such as NKG2D, and the resulting high level of hypoxia-inducible factor 1 alpha (HIF-1 alpha) is correlated with a worse prognosis in NSCLC (97). This is possibly due to adenosine and lactate accumulation that block NK activation and cytotoxicity, increasing the number of regulatory inhibitory cells like Tregs and myeloid-derived suppressor cells (MDSCs) (97). Natural Polyphenols can exerts antitumor activity and circumvent anti-PD-1 resistance (98). We have investigated the effects of a polyphenol rich olive mill wastewater derived polyphenols on the immune-microenvironment of lung cancer to overcome resistance (99).

## 4 Natural Killer Cells as a Potential Predictive Biomarker for Immunotherapy in Non-Small Cell Lung Cancer

### 4.1 Natural Killer Cells and Anti-PD-1/PD-L1

**Figure 2:**
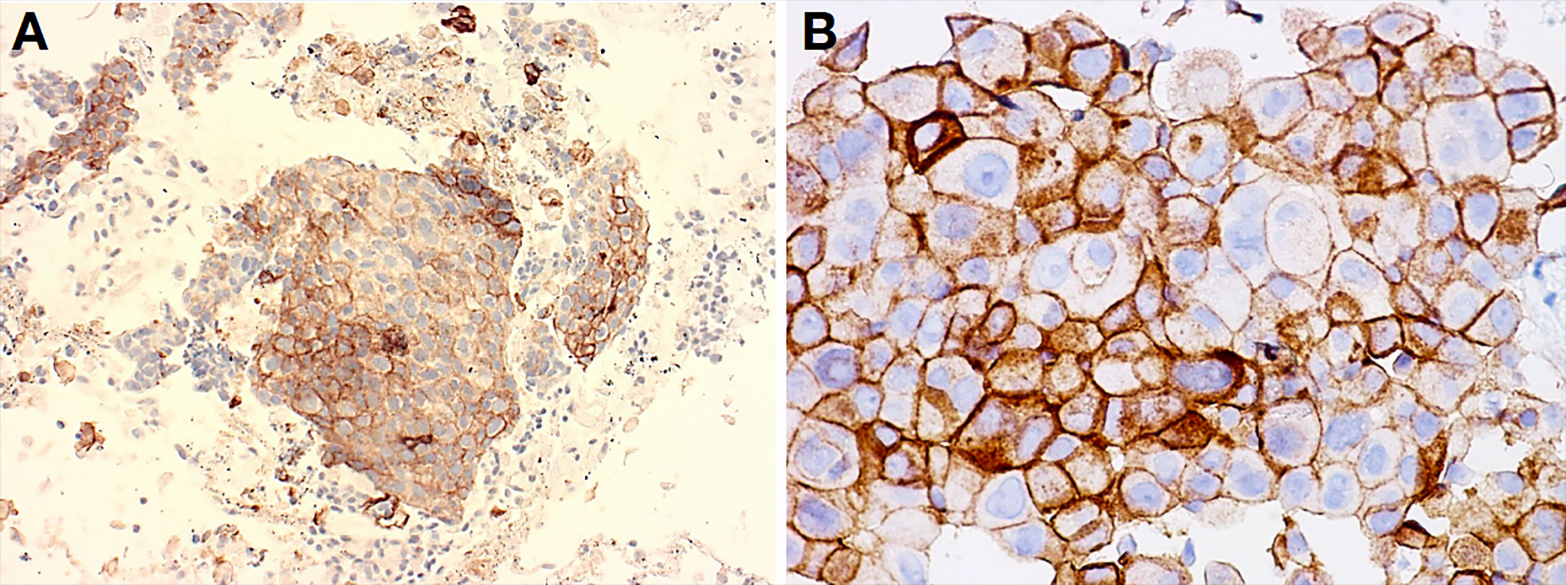
Lung adenocarcinoma obtained with EBUS-TBNA procedure **(A)** and pleural effusion (cell block) **(B)**. These two cases of metastatic adenocarcinoma to a mediastinal lymph node **(A)** and pleura cavity **(B)** featured solid-clumped patterns of growth, which turned out positive for TTF1 and negative for p40, thus confirming the correct subtyping as required by the current WHO guidelines (not shown). When tumors were made to react with antibodies to PD-L1 within companion kits, clusters of tumor cells unequivocally revealed membrane decoration in more than 50% of them, thus suggesting amenability of immunotherapy. Clone Agilent-Dako 22C3 was developed in Autostainer Link 48, with original magnification at ×400 **(A)**, while Ventana-Roche clone SP263 was developed in BenchMark Ultra IHC at ×200 **(B)**.

**Figure 3:**
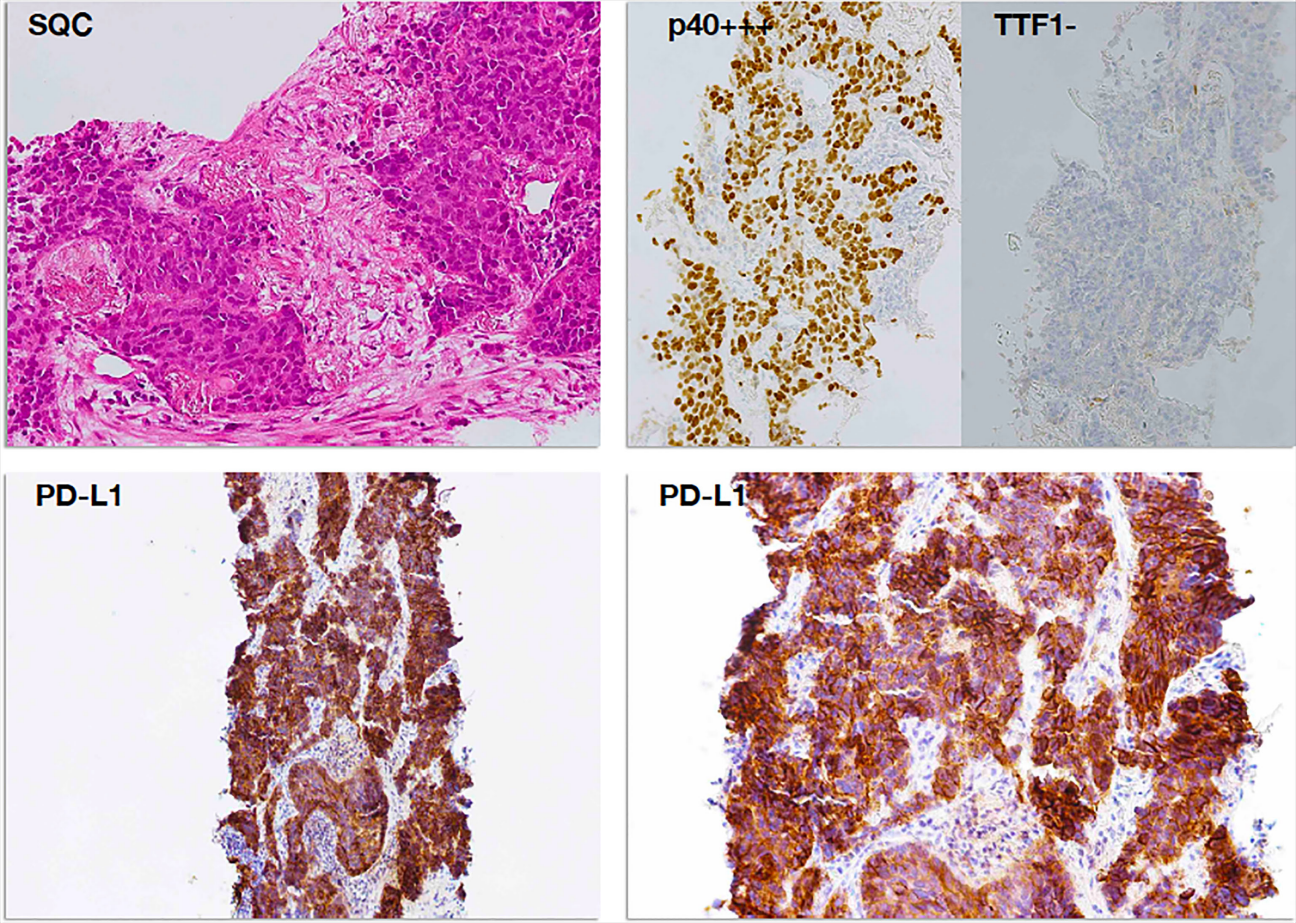
PD-L1 immunostaining in a case of squamous cell carcinoma (SCC). Poorly differentiated squamous cell carcinoma (top left panel, H&E staining) was readily subtyped by means of immunoreactivity for p40 (p40+++) and negativity for TTF1 (TTF1−), according to the current WHO guidelines (top right panel). Diffuse and intense membrane immunostaining for PD-L1 was observed in tumor cells, already evident at lower magnification (×100, bottom left panel) and then confirmed at higher magnification (×200, bottom right panel) as membrane decoration in over 95% of tumor cells.

ICIs targeting the PD-1/PD-L1 axis are now a milestone in the treatment of NSCLC (**Figures 2**, **3**), both as a single agent and in combination therapies (5, 6, 10–12, 100). In the lung, there are two main subtypes of NSCLC, namely, adenocarcinoma and SCC, for which immunotherapy may be a valuable strategy for the treatment of driver-negative metastatic patients (101). The WHO guidelines have emphasized the importance of the precise subclassification of NSCLC in both resection specimens and small-sized diagnostic material, the utility of immunohistochemical biomarkers in the accurate diagnosis and subclassification of NSCLC, and the critical role of molecular characterization for targeted therapy (102). The use of a marker of adenocarcinoma, such as thyroid transcription factor 1 (TTF1), and a marker of squamous cell differentiation, such as p40, is recommended as a two-hit, sparing-material minimalist antibody panel approach for reliably subtyping tumors (103). In clinical practice, after subtyping, the suitability for immune checkpoint axis-based immunotherapy is usually evaluated by means of the immunohistochemical detection of PD-L1 on tumor cells, which turned out to be a potential predictor of response to inhibitors especially when it is higher than 50% (104). The immunohistochemical reaction to PD-L1, which is quantified as the percentage of immunolabeled cells on the membrane independent of its completeness or intensity (realizing the so-called tumor proportion score) (104), is shown in **Figure 2** for adenocarcinoma and **Figure 3** for SCC. In clinical practice, it is usual to substitute the immune checkpoint axis by means of the immunohistochemical detection of PD-L1 on cancer cells, which is a potential predictor of response to inhibitors, especially when it is higher than 50 (104). PD-L1 expression is highly variable among different malignancies, and it can be very heterogeneous even in the same tumor, with different levels of expression depending on the area considered, as shown in **Figures 2** and **3** (104). Recent studies demonstrated that NKs play a crucial role in tumoral response to ICIs. PD-1 is expressed by NKs in PB and TME in multiple cancers, including lung cancer (90–92). After binding to PD-L1, PD-1-positive NKs become unpaired in mouse and human models, suggesting that downregulation of this pathway plays an important role not only in T cells but also in other immune cells of the TME. By PD-1/PD-L1 blockade, ICIs partially restore the normal NK activity, highlighting the therapeutic and predictive potential of PD-1-positive NKs (105–107). Interestingly, PD-1/PD-L1 blockade may also influence NK activity by inducing a Treg downregulation, Treg inhibit NK function and survival through TGF-beta release (105, 106). Several mouse models showed that the therapeutic effect was NK-dependent in MHC-deficient tumors, while in MHC- expressing tumors (T-cell sensitive), NK depletion has the same effect as CD8^+^ T-cell depletion (105). Accordingly, mice lacking both T cells and NKs do not develop any response after PD-1 blockade (108). NKs can influence response to ICIs also triggering antibody-dependent cell-mediated cytotoxicity (ADCC) against cancer cells in *in vitro* models, as the Fc gamma receptor (Fcγ) on NKs is an integral part of the ADCC mechanism (109). Furthermore, NKs can stimulate the migration and survival of CD141^+^ dendritic cells (DCs) *via* chemokines and cytokine production (i.e., CCL5, XCL-1, or lymphotactin, FLT3-Ligand) (110). Finally, NKs release a great amount of IFN-γ, which can induce PD-L1 expression on tumor cells, increasing sensitiveness to ICI (110).

In conclusion, PD-1 is an important regulator of NK function, and its blockade was shown to enhance NK activity leading to increased tumor control. Moreover, NKs were shown to play a role in response to PD-1–PD-L1-based therapies against tumors both sensitive and resistant to T cell-mediated cytotoxicity.

Due to their central role in immune response, NKs may be involved in predicting tumor response to immunotherapy. An NK-related gene expression profile performed on NSCLC patients treated with nivolumab or pembrolizumab was found to be correlated to treatment response and PFS (111). In another prospective trial of nivolumab in NSCLC, patients who achieved clinical benefit showed higher baseline functionally active circulating NKs compared to non-responders. Circulating NK numbers progressively increased during PD-1 blockade in responders, counterbalanced by a reduction in circulating Tregs (112).

Furthermore, stratification of patients by quantification of NK receptors from blood samples could be a useful prognostic and predictive tool: low expression of Natural Cytotoxicity Triggering Receptor (NCR) 1 and 3 correlated with worse prognosis in NSCLC and PD-L1-positive (>5%) patients, and low NCR3 expression correlated with the worse outcome to anti-PD1 (113).

As it is well known that NKs are primary producers of IFN-γ, the best overall response rate (ORR) to nivolumab has been reported in advanced NSCLC patients with higher expression of the IFN-γ target gene (114).

An exploratory analysis of the phase II randomized POPLAR trial showed that NSCLC patients with high T-effector–IFN-γ-associated gene expression had improved OS with atezolizumab compared to docetaxel (115). Circulating levels of IFN-γ might reflect the activation of the IFN-γ signaling and could be an easy tool to monitor patients during treatment. In a prospective study on 26 NSCLC patients treated with pembrolizumab or nivolumab, increased blood levels of IFN-γ and in addition other cytokines (TNF-alpha, IL-1β, IL-2, IL-4, IL-6, and IL-8) at the time of diagnosis and 3 months after the start of the treatment were significantly correlated with improved response to immunotherapy and prolonged OS, while no correlation with PD-L1 expression was found (116). However, robust data on the specific contribution of NKs in response to anti-PD-1/PD-L1 drugs in NSCLC are limited. Further prospective studies are needed to assess the predictive role in this context, and no data are available on the modulation of NKs under concomitant chemotherapy and immunotherapy.

### 4.2 Natural Killer Cells and Anti-CTLA-4

The CTLA-4 is abundantly expressed by Tregs and, upon stimulation, by cytotoxic T cells (9, 100). While interest in the role of innate immunity in anti-PD-1/PD-L1 therapy is growing, little is known about NKs in CTLA-4-based therapies. Although under IL-2 stimulation murine NKs can exhibit CTLA-4 on their surface, this does not happen in humans (117, 118).

Anti-CTLA-4 seems to increase intratumor NK levels in melanoma murine models, positively affecting response, especially when treatment was combined with IL-2 (119). In melanoma patients, high levels of intratumor NKs in pretreatment tumor samples were correlated to improved outcomes of anti-CTLA-4 (120), and survival rate was correlated with low levels of IL-15 in the serum. In PB of patients with malignant pleural mesothelioma (MPM), a reduction of CD56^dim^ effector NKs was observed as compared to healthy controls, but the levels of these cells increased after therapy with tremelimumab, an anti-CTLA-4 monoclonal antibody (mAb) (121). Similar results were reported in melanoma patients after treatment with ipilimumab (122).

Treg downregulation upon anti-CTLA-4 therapy can result in reduced Treg-mediated NK inhibition (123). Taken together, these data suggest a possible interplaying role between CTLA-4 blockade and NKs. These observations might be useful, as combination therapy of nivolumab, ipilimumab, and standard chemotherapy has recently been approved in clinical practice as a first-line option for metastatic NSCLC. A combination of multiple checkpoints might overcome NK resistance to a single agent and restore NK immunity. However, to date, no data are available in this field.

## 5 Natural Killer Cells as a Target of New Therapeutic Strategies

Resistance to anti-PD-1/PD-L1 and anti-CTLA-4 ICIs can occur during treatment, as a result of the exhaustion of immunological targets or the activation of alternative pathways, under pressure and dynamic changes of the TME. Thus, there is an urgent need for the development of new pharmacological agents able to block these evasion mechanisms. As for PD-1/PD-L1 and anti-CTLA 4, blocking other cancer-dependent inhibitory pathways, either through single agents or in combinations with other ICIs, is one of the most studied strategies to obtain disease control. Mobilization of NKs, which can coordinate the anticancer response together with T cells, may also be a promising therapeutic strategy. As reported above, NKs have a crucial role in tumor response and are also possibly implicated in response to immunotherapy. Many efforts have been made to target NKs as therapeutic agents. NK immunotherapy can be approached from two directions: the activation of endogenous NKs currently circulating or resident within normal or tumor tissues or the administration of activated autologous or allogeneic NKs. **Tables 1** and **2** summarize ongoing clinical trials with ICIs and adoptive or chimeric antigen receptor (CAR)-NK therapy in NSCLC. Most of the targets that are being explored by new ICIs are expressed on NKs.

**Table 1:** Current clinical trials with checkpoint inhibitors in NSCLC.

Identifier	Drug	Phase	Study design	Setting	Status
**TIGIT**					
**NCT04294810** **SKYSCRAPER-01**	Tiragolumab	III	Tiragolumab + atezolizumab vs. placebo + atezolizumab	Untreated advanced NSCLC PD-L1 pos.	Active, recruiting
**NCT03563716** **CITYSCAPE-01**	Tiragolumab	II	Tiragolumab + atezolizumab vs. placebo + atezolizumab	Untreated advanced	Active, not recruiting
**NCT04746924**	Ociperlimab	III	Ociperlimab + tislelizumab vs. pembrolizumab in PD-L1 ≥ 50%	Untreated advanced PD-L1 pos.	Active, recruiting
**NCT05102214**	HLX301	I/II	HLX301 (bi-specific: TIGIT and PD-1) single-arm multicohort	Previously treated solid tumors	Active, recruiting
**KIR**					
**NCT01714739**	Lirilumab	I/II	Lirilumab + nivolumab or nivolumab and ipilimumab	Pretreated solid tumors	Completed
**NCT03347123**	Lirilumab	I/II	Lirilumab + nivolumab + epacadostat	Pretreated solid tumors	Completed
**TIM-3**					
**NCT03708328**	RO7121661	I	RO7121661 (Bi-specific: TIM-3 and PD-1) single arm dose escalation phase + expansion cohort	Advanced solid tumors	Active, recruiting
**NCT03744468**	BGB-A425	I/II	BGB-A425+ tislelizumab multicohort	Stage III-IV NSCLC PD-L1 positive	Active, recruiting
**LAG-3**					
**NCT02966548**	Relatlimab	I	Relatlimab + nivolumab	Pretreated, metastatic solid tumors	Active, not recruiting
**NCT01968109**	Relatlimab	II	Relatlimab + nivolumab	Solid tumors (I or II line NSCLC)	Active, not recruiting
**NCT03459222**	Relatlimab	I/II	Relatlimab + nivolumab + ipilimumab	Solid tumors	Not recruiting
**NCT03625323**	IMP321 (eftilagimod alpha)	II	Eftilagimod alpha + pembrolizumab	I or II line NSCLC	Active, not recruiting
**NCT04623775**	Relatlimab	II	Relatlimab + nivolumab + Chemotherapy vs. nivolumab + chemotherapy	First-line stage IV NSCLC	Active, recruiting
**NCT04205552**	Relatlimab	II	Relatlimab + nivolumab vs. nivolumab	Neoadjuvant stage IB–IIIA NSCLC	Active, recruiting
**NKG2A**					
**NCT03822351**	Monalizumab	II	Durvalumab/monalizumab/oleclumab Following chemo-radiotherapy	Stage III NSCLC	Active, not recruiting
**NCT05061550**	Monalizumab	II	Durvalumab/monalizumab/oleclumab	Neo/adjuvant stage IB–IIIA NSCLC	Active, not recruiting

The table shows phase I–III ongoing clinical trials on new checkpoint inhibitors acting on NK.

NK, natural killer cell; NSCLC, non-small cell lung cancer.

**Table 2:** Current clinical trials on adoptive and CAR-NK therapy in NSCLC.

Identifier	Type of NK	Patient number	Phase	Drugs	Setting	Current status
NCT04990063	Autologous	20	I	Natural killer cells (NKs) and gamma delta T cells (γδT cells) + chemotherapy	Advanced NSCLC	Active, recruiting
NCT02843204	Allogenic	109	I/II	Allogenic NK + pembrolizumab	Advanced pretreated NSCLC	Completed
NCT02118415	Autologous	90	II	Hsp70-peptide TKD/IL-2 activated, autologous NKs	Maintenance therapy, unresectable stage III NSCLC after chemo-radiotherapy	Suspended
NCT04616209	Allogenic	24	I/II	Allogeneic PB103 and standard cancer treatment	Stage IIIB–C/IV	Active, recruiting
NCT04872634	Allogenic	24	I/II	SNK01 + chemotherapy ± cetuximab	Advanced NSCLC, pretreated with TKI	Active, recruiting
NCT03656705	CAR-NK	5	I	Chimeric costimulatory converting receptor (CCCR)-modified NK92 cells in previously treated advanced non-small cell lung carcinoma	Advanced pretreated NSCLC	Enrolling by invitation
NCT03841110	Allogenic	37	I	FT500 (allogeneic, iPSC-derived NK) monotherapy or plus pembrolizumab/nivolumab/atezolizumab	Advanced pretreated NSCLC	Active, recruiting
NCT04440735	BIKE	100	I	DSP107(SIRPα-4-1BBL) + Atezolizumab	Advanced refractory NSCLC	Active, recruiting
NCT04050709	CAR-NK	16	I	PD-L1 t-haNK	Pretreated solid tumors	Active, not recruiting

The table summarizes the ongoing trials on genetically modified and non-genetically modified adoptive cell therapy with NKs.

NK, natural killer cell; NSCLC, non-small cell lung cancer; TKI, tyrosine kinase inhibitor.

### 5.1 Inhibitors of Natural Killer Cell Receptors in Non-Small Cell Lung Cancer

**Figure 4:**
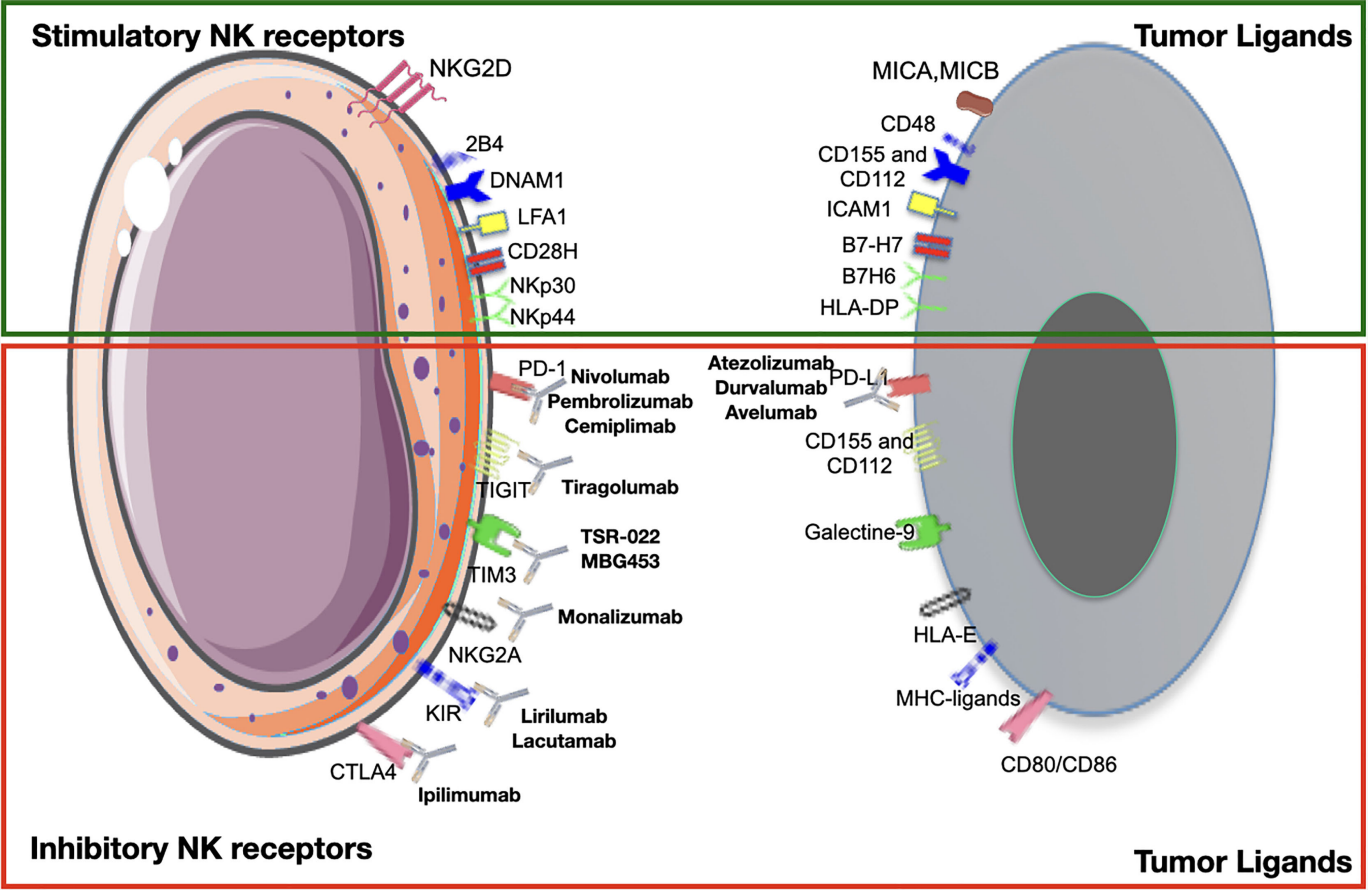
Activating and inhibitory receptors on natural killer cells (NKs) and their ligands on tumor cells. Checkpoint inhibitors bind to inhibitory receptors on NKs, preventing the link with their ligands on tumor cells and vice versa. Here are reported the major pathways and monoclonal antibodies currently used in clinical practice or under evaluation in clinical trials.

As exhausted TI-NKs or TA-NKs express inhibitory receptors on their cell surface, one strategy to overcome resistance is to target in order to restore NKs to their antitumoral activity (**Figure 4**).

#### 5.1.1 Anti-KIR

Monoclonal antibodies targeting KIR inhibitory receptors KIR2DL1–3 have been developed. They mimic the “missing-self” response by blocking the interaction between KIR2DL and the natural ligand HLA-C. Lirilumab is a fully humanized IgG4 that binding with high affinity to KIR2DL1–3 receptors, expressed in about half of TI-NKs and TA-NKs, blocks the interaction with HLA-C. Lirilumab was first investigated on hematologic malignancies (124), but it showed initial efficacy and a favorable safety profile also in a phase I basket trials comprising various tumor types (breast, ovarian, pancreatic, and endometrial cancers) (125). A combination of lirilumab and nivolumab showed promising activity in the SCC of the head and neck (126). Lirilumab is under investigation in various solid tumors, including NSCLC, alone or in combination with nivolumab and epacadostat in a multicohort phase I–II study (NCT03347123).

#### 5.1.2 Anti-NGK2A

NKG2A is another inhibitory receptor expressed on the NK surface (76, 77). After interaction with a non-classical MHC class I molecule, it forms heterodimers with CD94 leading to the activation of inhibitory intracellular signals. As reported above, NKG2A is overexpressed on NKs in many types of cancers and is related to worse prognosis and immunotherapy resistance. This has encouraged the development of a specific monoclonal antibody blocking this pathway, called monalizumab (76, 77). Furthermore, when combined with anti-PD-1, monalizumab can stimulate also CD8^+^ T-cell antitumor activity (76, 77). Monalizumab is currently under investigation in combination with durvalumab in solid tumors and in particular in NSCLC (NCT02671435, NCT03833440).

#### 5.1.3 Anti-TIM-3

Preclinical evidence suggests that TIM-3 blockade alone or in combination with PD-1 inhibitors can reverse the functional impairment of TIM-3+ T cells (127, 128). An increase in TIM-3+ circulating NKs has been reported in lung cancer, associated with immune suppressive TME, NK killing activity inhibition, and the more aggressive disease form, suggesting that it could be a possible therapeutic target in NSCLC (84). Preclinical data demonstrated that TIM-3 blockade is effective not only in counter-modulating dysfunctional CD8^+^ T cells and Tregs but also in restoring NK cytotoxic activity and TNF-alpha and IFN-γ production in lung cancer (83). Therapeutic TIM-3 antibodies are currently being evaluated in phase I trials either as single-agent treatment or in combination therapy [NCT03744468, NCT03708328]. Furthermore, a bi-specific antibody targeting both PD-1 and TIM-3, AZD7789, has recently been developed and is currently under investigation in a phase I trial in patients with different solid tumors, including lung cancer [NCT03708328].

#### 5.1.4 Anti-LAG3

Although the specific role of LAG-3 in human NKs is still unclear, the therapeutic blockade of this checkpoint receptor remains appealing due to its interaction with both NK and T cells, particularly in combination with PD-1 inhibitors. Certain clinical-grade inhibitors (IMP321, BMS-986016) are currently under investigation in ongoing phase I and II trials (129).

#### 5.1.5 Anti-TIGIT

TIGIT is an inhibitory receptor expressed on CD8^+^ T cells, Tregs, and NKs and has gained increasing attention as a promising novel pharmacological target for cancer immunotherapy. Binding to its ligands CD155 and CD112 (or nectin-2) expressed by tumor cells and antigen-presenting cells in the TME, TIGIT induces anergy of T cells and NKs, immune suppression, and tumor escape (130). The combination of the anti-TIGIT antibody, tiragolumab, with atezolizumab showed encouraging results in NSCLC. In preclinical models, the combination of anti-TIGIT and anti-PD-L1 synergistically improved tumor control and survival (131). The randomized phase II CITYSCAPE compares the first-line treatment with tiragolumab plus atezolizumab with atezolizumab alone in metastatic NSCLC patients, stratified for histology and selected for PD-L1 expression (132). The combination of tiragolumab and atezolizumab significantly improved ORR (37% versus 21%) and PFS (median PFS (mPFS) 5.42 versus 3.58), independently from the histology, with a greater magnitude benefit in patients with PD-L1 > 50% (133). The blockade of TIGIT could be an interesting chemo-sparing strategy; however, longer follow-up and phase III trials are required.

### 5.2 BiKEs and TriKEs

Bi- and Tri-specific T-cell engagers (BiTEs and TriTEs) are bi- or tri-valent antibodies constituted by two or three single-chain Fc fragments, respectively, that create a link between T cells and tumor cells (134). T cells lack Fcγ receptors, so normal monoclonal antibodies are not able to directly recruit T cells (135). Thanks to their two or three chains, BiTEs and TriTEs can recognize both one or two tumor antigens and one CD3 molecule, associated with the TCR, at the same time resulting in T-cell activation (136). This is an intriguing strategy to re-activate exhausted T cells induced by long-term exposure to tumor antigens. More recently, the same mechanism has been designed for bi- or tri-specific killer−cell engagers (BiKEs and TriKEs) to recruit and activate NKs in the TME and to promote tumor lysis. These molecules are built up by two (BiKEs) or three (TriKEs) single-chain variable fragments (scFv) with different heavy and light antibody chains connected through short peptide linkers (137). These can be considered “NK cell adaptors”; they usually target an activating receptor, like NKp46 and CD16 on NKs and a tumor antigen, such as CD19, CD20, or endothelial growth factor receptor (EGFR) and Fc fragments (138). Compared to monoclonal antibodies, BiKEs and TriKEs present some important advantages, such as higher biodistribution, due to their small size, lower immunogenicity, and great flexibility (139). AFM24 is a bispecific EGFR/CD16A innate cell engager antibody that has shown preclinical activity in controlling tumor growth in *in vitro* and mouse models of EGFR-positive tumors, independently from the presence of EGFR mutation (140). TriKEs have the ulterior advantage of targeting two different molecules, preventing an eventual downregulation of one selected molecule on target tumor cells (137). A new generation of TriKEs and TetraKEs (with four functional domains) has been designed to incorporate an IL-15 moiety, with the aim of promoting NK activation, *in vivo* persistence, and proliferation. However, most of these agents are only in preclinical development, and further studies are needed before testing them in a clinical setting.

### 5.3 Adoptive Natural Killer Cell Therapy

#### 5.3.1 Non-Genetically Modified

An alternative approach to the systemic activation of NKs is to directly introduce activated NKs into patients. This adaptive transfer of NKs is the most direct way to restore and improve the function of the immune system. NKs can be autologous or allogenic as derived from PB mononuclear cells (PBMCs) or stem cells (umbilical cord blood and embryonic stem cells) or NK lines. Following isolation, NKs can be activated by exposure to cytokines or other stimulating factors or by genetically engineered manipulation (141, 142). To assess the feasibility of autologous NK transfer, a study was designed in patients with advanced NSCLC and treated with a combination of docetaxel and *ex vivo* expanded autologous NK. However, it failed to demonstrate a real therapeutic benefit of the combination, probably due to poor NK activity *in vivo* (143). Even though autologous NKs can be efficiently expanded and activated *in vitro*, the unsuccessful result of this study suggests that this approach is probably not a feasible treatment modality (42, 143, 144). Compared to autologous NKs, allogeneic NKs have a longer persistence *in vivo*, which corresponds to an improved response to treatment but is burdened with a higher risk of graft-versus-host disease (145). Adaptive allogenic or alloreactive transfer results in MHC-I and KIR ligand mismatch and efficient immune response, as reported first in AML (146). Following AML, clinical studies testing the adaptive transfer of mismatched alloreactive NKs as a form of immunotherapy showed low toxicity and initial therapeutic efficacy also in solid tumors including NSCLC (147, 148). In advanced NSCLC, repetitive infusions of alloreactive donor NKs resulted in encouraging disease control in many patients, highlighting its potential use in this setting (149). Furthermore, the combination of allogenic adaptive NK therapy with pembrolizumab led to improved OS and PFS (median OS (mOS) 15.5 vs. 13.3 months; mPFS 6.5 vs. 4.3 months; p < 0.05) as compared to pembrolizumab alone in pretreated advanced NSCLC patients. The survival advantage was particularly evident in PD-L1-positive patients (>50%) (149).

To expand the therapeutic use of alloreactive NKs, human NK lines have been generated as a renewable source of NKs. The human NK line NK-92 (150) is highly cytotoxic against a variety of cancer types, and it is under investigation also in phase I trials in solid tumors, such as melanoma (151, 152). The NK-92 cell line has been used as a source of NKs for adaptive transfer and modified for improved efficacy and target specificity, with genetic manipulation or cytokine activation prior to adaptive transfer. In a phase I basket trial, infusion of NK-92 cells was particularly active in patients with lung cancer patients: three of four small cell lung cancer (SCLC) and NSCLC patients in the study have tumor response according to Response Evaluation Criteria in Solid Tumors (RECIST) criteria or long-lasting disease control with the adaptive transfer of IL-2 activated NK-92 cells (153). A hypothesis about this particular sensitivity in lung cancer patients is that NKs reside in the lung prior to circulating following intravenous administration (142). These could represent an intriguing strategy to develop in dedicated clinical trials.

#### 5.3.2 Chimeric Antigen Receptor–Natural Killer Cells

Genetically modified NKs present enhanced specificity and activity against the target. One of these methods that are founding increasing interest is the construction of CARs based on NKs instead of T lymphocytes (154). CAR-T cells are now widely used in clinical practice, mostly in hematological malignancies, but this technology has also been applied to macrophages and NKs to enhance efficacy and limit possible toxicity (155). In fact, CAR-NK administration is not associated with the development of cytokine release syndrome, neurotoxicity or graft-versus-host response, and other side effects, which makes it a very attractive therapeutic option (156).

The sources of NKs usable for CAR-NK are the same as for adaptive therapy: PB or umbilical cord NKs or cell lines, such as NK-92. Recently, a CAR-NK was created using NK-92-derived cell lines carrying on the surface the immune checkpoint anti-B7-H3: in xenograft models, it showed significant inhibition of tumor growth and increased survival, providing a proof of concept for its development in the clinical setting (157). Another interesting new chimeric costimulatory converting receptor was built up by modified NK92 and constituted by the extracellular domain of PD-1, transmembrane and cytoplasmatic NKG2D domain, and cytoplasmic domain of the TNF receptor superfamily member 4−1BB (TNFRSF9/CD1377). It is able to counteract the immunosuppressive action of PD-1 and showed preclinical *in vitro* anti-humoral activity against human lung cancer H1299 cells (158). Delta-like ligand 3 (DLL3) is overexpressed in most SCLC and may be used as a target for CAR-NKs therapy. In a recent study, DLL3-positive SCLC cell lines have been cocultured with DLL3-CAR-NK-92, and the construct was proved to have a high cytolytic effect (158). This report explored the potential in the treatment of SCLC. Furthermore, the DLL3-CAR NK-92 showed improved cytotoxicity also against lung metastasis in tumor models with good tolerance and in subcutaneous tumor models of SCLC (158).

## Conclusion

Cancer treatment with ICIs of PD-1/PD-L1 and CTLA-4 is widely used in clinical practice, but, unfortunately, it shows limited efficacy in a variety of patients due to secondary resistance or non-response. In physiological conditions, NKs play an important role in the immune response against the tumor. However, following neoplastic transformation, cancer cells and TME act by modulating NK functions inducing the switch toward a pro-tumor phenotype (42, 144). TME can influence the treatment response and effectiveness of ICIs, and a growing amount of evidence suggest that NKs can act as a predictor as well as a prognostic factor (42, 144). NKs have been shown to play a crucial role in metastatic tumor surveillance in both NSCLC and SCLC. In recent years, the application of NK and CAR-NK immunotherapy has brought significant progress in the field of cancer therapy, with the latest clinical trials showing tremendous potential (155). Although the clinical focus of NK therapy is largely hematopoietic malignancies, conceivable progression of NK immunotherapy in the treatment of lung cancer has also emerged.

The lung cancer cells and the TME can polarize NKs into pro-inflammatory, pro-angiogenic decidual-like subsets (42, 155). Therefore, although the application of NK therapy as a standalone agent or in combination with other therapeutic modalities is a rapidly evolving field that is producing promising results, the possibility of turning the tumor and TME into a non-lytic phenotype has to be taken into account. The findings summarized in this review have yet to be fully confirmed in more in-depth clinical settings, but they highlight a potential diagnostic and therapeutic modality in a field with limited therapeutic options and an invariably low survival rate.

Curbing NK pro-inflammatory switch in cancer is pivotal in the success of immunotherapy with ICI.

## Author Contributions

Conceptualization: AA, MG, and RR. Writing—original draft preparation: MG and AA. Writing—review and editing: AA, RR, DN, VC, MB, and GP. Figures: AA, MG, DN, GP, and MB. All authors have read and agreed to the published version of the manuscript.

## Funding

DM was the recipient of the Ministero della Salute COVID-2020-12371849 and the Italian Ministry of University and Research PRIN 2017 grant 2017NTK4HY. AA is the recipient of a research contract in progress from Stazione Zoologica (ADViSE Project; PG/2018/0494374) and Azienda Agricola Fattoria La Vialla, Castiglion Fibocchi, Arezzo (Italy). This work has also been supported by the Italian Ministry of Health Ricerca Corrente—IRCCS MultiMedica to MG, GP, and RR and IRCCS IEO to AA.

## Conflict of Interest

The authors declare that the research was conducted in the absence of any commercial or financial relationships that could be construed as a potential conflict of interest.

## Publisher’s Note

All claims expressed in this article are solely those of the authors and do not necessarily represent those of their affiliated organizations, or those of the publisher, the editors and the reviewers. Any product that may be evaluated in this article, or claim that may be made by its manufacturer, is not guaranteed or endorsed by the publisher.
